# Tyrosine Kinase Inhibitor Activity in Patients with NSCLC Harboring Uncommon *EGFR* Mutations: A Retrospective International Cohort Study (UpSwinG)

**DOI:** 10.1093/oncolo/oyac022

**Published:** 2022-03-11

**Authors:** Sanjay Popat, Te-Chun Hsia, Jen-Yu Hung, Hyun Ae Jung, Jin-Yuan Shih, Cheol Kyu Park, Seung Hyeun Lee, Tatsuro Okamoto, Hee Kyung Ahn, Yong Chul Lee, Yuki Sato, Sung Sook Lee, Celine Mascaux, Hasan Daoud, Angela Märten, Satoru Miura

**Affiliations:** 1 Lung Unit, Royal Marsden National Health Service Foundation Trust, London, UK; 2 The Institute of Cancer Research, London, UK; 3 Department of Respiratory Therapy, China Medical University, Taichung, Taiwan; 4 Department of Internal Medicine, Division of Pulmonary and Critical Medicine, China Medical University Hospital, Taichung, Taiwan; 5 Faculty of Medicine, College of Medicine, Kaohsiung Medical University, Kaohsiung, Taiwan; 6 Division of Hematology-Oncology, Department of Medicine, Samsung Medical Center, Sungkyunkwan University School of Medicine, Seoul, South Korea; 7 Department of Internal Medicine, National Taiwan University Hospital, No. 7, Chung-Shan South Road, Taipei, 100, Taiwan; 8 Department of Internal Medicine, Chonnam National University Hwasun Hospital, Chonnam National University Medical School, Hwasun, South Korea; 9 Division of Pulmonary and Critical Care Medicine, Department of Internal Medicine, Kyung Hee University Medical Center, Kyung Hee University School of Medicine, Dongdaemun-gu, Seoul, South Korea; 10 Department of Thoracic Oncology, National Hospital Organization Kyushu Cancer Center, Fukuoka, Japan; 11 Division of Medical Oncology, Gachon University Gil Medical Center, Incheon, South Korea; 12 Department of Internal Medicine, Research Center for Pulmonary Disease, Biomedical Research Institute of Jeonbuk National University Hospital, Jeonbuk National University Medical School, Jeonju, South Korea; 13 Department of Respiratory Medicine, Kobe City Medical Center General Hospital, Hyogo, Japan; 14 Inje University Haeundae Paik Hospital, Inje University College of Medicine, Busan, South Korea; 15 Pulmonology Department, University Hospital of Strasbourg, 67091 Strasbourg Cedex, France; 16 Université de Strasbourg, Inserm UMR_S 1113, IRFAC, Laboratory Streinth (STress REsponse and INnovative THerapy against cancer), ITI InnoVec, 67200 Strasbourg, France; 17 Boehringer Ingelheim International GmbH, Ingelheim am Rhein, Germany; 18 Department of Internal Medicine, Niigata Cancer Center Hospital, Niigata, Japan

**Keywords:** EGFR, uncommon *EGFR* mutations, afatinib, osimertinib, gefitinib, erlotinib

## Abstract

**Background:**

Epidermal growth factor receptor tyrosine kinase inhibitors (EGFR TKIs) are standard of care for patients with *EGFR* mutation-positive non–small-cell lung cancer (NSCLC) with common mutations (Del19 or L858R); however, 7%-23% of NSCLC tumors harbor uncommon *EGFR* mutations. These mutations are highly heterogeneous, and developments in detection techniques are helping to identify mutations with little or no clinical data.

**Patients and Methods:**

In this retrospective, global, multi-center study (NCT04179890), existing health records were identified for consecutive EGFR TKI-naïve patients with uncommon *EGFR* mutations (T790M, ex20ins, major uncommon [G719X, L861Q, or S768I], or “other” mutations; compound mutations) treated with erlotinib, gefitinib, afatinib, or osimertinib in first or second line. Endpoints included time-to-treatment failure (TTF), objective response rate (ORR), and overall survival (OS).

**Results:**

Overall, 246 patients (median age: 69.5 years; Asian: 84%) were included from 9 countries. Most patients (92%) received an EGFR TKI as first-line therapy; 54%, 43% and 3% received afatinib, first-generation TKIs, and osimertinib, respectively. Median TTF and OS with EGFR TKIs were 9.9 and 24.4 months; ORR was 43%. In patients treated with first-line chemotherapy (*n* = 20), median TTF and ORR were 6.6 months and 41%. Outcomes were most favorable in patients with major uncommon or compound mutations. Overall, TTF was 11.3 months with afatinib and 8.8 months with first-generation EGFR TKIs across mutation categories. In most mutation categories, median OS was >2 years.

**Conclusion:**

In a real-world setting, EGFR TKIs were the preferred treatment option in patients with uncommon *EGFR* mutations; strongest outcomes were seen in patients with major uncommon and compound mutations.

## Teaser

This article reports partial findings of the UpSwinG study, focusing on a cohort of patients with tumors harboring uncommon EGFR mutations who received first- or second-line EGFR tyrosine kinase inhibitor treatment.

**Implications for Practice**

This retrospective study provides further “real-world” evidence of the activity of EGFR TKIs against certain uncommon *EGFR* mutations. Epidermal growth factor receptor TKIs should be considered as treatment options for patients with major uncommon (G719X, L861Q, or S768I), compound and some other uncommon mutations. Optimal treatment of *EGFR* mutation-positive NSCLC in everyday practice requires improvements in pathology reports, with more emphasis on implementation of NGS methodology and precise definition of mutations.

## Introduction

Over the past decade, first-line treatment of patients with epidermal growth factor receptor (*EGFR)* mutation-positive non–small-cell lung cancer (NSCLC) has been revolutionized with the development of first-generation (gefitinib and erlotinib^1-6^), second-generation (afatinib and dacomitinib^7-10^), and third-generation (osimertinib^11^) EGFR tyrosine kinase inhibitors (TKIs). All of these agents were approved based on robust clinical trials that demonstrated significantly improved progression-free survival (PFS)^1-11^ and, in some cases, overall survival (OS)^12-14^ versus chemotherapy or first-generation EGFR TKIs. However, as most of these trials were limited to patients with the so-called “common” *EGFR* mutations (exon 19 deletions [Del19] and the L858R mutation in exon 21) few prospective data are available to inform treatment decisions for the estimated 7%-23% of *EGFR* mutation-positive NSCLC tumors that harbor uncommon *EGFR* mutations.^15-22^ To date, only 4 randomized trials of EGFR TKIs have included a small number of patients with uncommon mutations: IPASS (gefitinib^4^), NEJ002 (gefitinib^5^), and LUX-Lung 3 and 6 (afatinib^7,8^).

Given the ongoing implementation of sensitive mutation detection methodologies, such as next-generation sequencing (NGS), and the increased use of circulating cell-free DNA NGS techniques, physicians are increasingly likely to encounter cases of *EGFR* mutation-positive NSCLC with uncommon mutations in everyday practice, for which the clinical evidence base is narrow.^23-25^ Therefore, more clinical data are required to inform treatment decisions in such cases. Uncommon *EGFR* mutations are highly heterogeneous but may be categorized into groups^26^: the most prevalent “major” uncommon mutations, G719X, L861Q, and S768I; exon 20 insertions, which are generally, but not always, insensitive to EGFR TKIs; de novo T790M; and “other” uncommon mutations comprising very rare mutations (point mutations, insertions, and deletions) across exons 18-21. Moreover, uncommon *EGFR* mutations can appear as part of a compound mutations (2 or more *EGFR* mutations within the same tumor). Recently, a classification system for uncommon *EGFR* mutations has been proposed based on the structural changes to the receptor: mutations which are distant from the ATP-binding pocket (classical-like mutations); mutations that occur in the hydrophobic core (eg, T790M); mutations that occur at the back of the ATP-binding pocket (eg, exon 20 insertions) or pocket volume-reducing (PVR) mutations that occur in the interior of the ATP-binding pocket or in the αc helix/A loop.^27^ However, this classification system would be difficult to apply in everyday clinical practice.

Available preclinical^23,28^ and in silico^29^ data indicate that uncommon *EGFR* mutations vary widely in terms of their sensitivity to different EGFR TKIs, with second- and third-generation TKIs generally demonstrating broader inhibitory activity across uncommon mutations than first-generation TKIs. Classical-like mutations appear to be widely sensitive to all EGFR TKIs, T790M-like mutations are sensitive to third-generation EGFR TKIs and PVR mutations are sensitive to second-generation EGFR TKIs.^27^ At present, more data exist for afatinib than other TKIs regarding clinical activity against specific uncommon mutations, supported by broader inclusion criteria in clinical trials. Recently, a database documenting outcomes of 693 patients with uncommon *EGFR* mutations treated with afatinib was published, comprising source data from LUX-Lung 3 and 6, compassionate-use and expanded-access programs, phase IIIb trials, retrospective trials and case studies.^26^ This analysis highlighted the activity of afatinib against the major uncommon mutations as well as many “other” uncommon mutations, compound mutations and certain exon 20 insertion variants.^26^ While fewer data are available for osimertinib, a recent phase II study, real-word data and case studies indicate that it also may be active against certain uncommon mutations.^30-32^ First-generation EGFR TKIs have also demonstrated modest activity against major uncommon mutations and some compound mutations in retrospective analyses.^33-38^ Despite these findings, further clinical data are required to help define personalized treatment strategies in individual patients depending on specific mutation type. Furthermore, another limiting factor is detection of uncommon mutations. Only by using appropriate methods and adequate clinical annotation can clinicians select the best treatment for the individual patient.

Here we describe the findings of a real-world, retrospective, global, and multi-center study (UpSwinG). The study used pre-existing data collected from the medical records of consecutive patients treated with EGFR TKIs (erlotinib, gefitinib, afatinib, or osimertinib) and comprised 2 cohorts. Cohort 1 included patients with tumors harboring uncommon *EGFR* mutations who received first- or second-line EGFR TKI treatment. Cohort 2 included patients with common *EGFR* mutations treated with sequential afatinib and osimertinib. The results from Cohort 1 are reported here. Cohort 2 is the subject of an additional analysis and will be presented separately.

## Methods

### Study Design

UpSwinG was a retrospective, global, multi-center study (NCT04179890) conducted across nine countries (UK, Taiwan, South Korea, Japan, France, Germany, Austria, Spain, Italy). Medical and electronic health records of consecutive patients treated in a real-world practice who met the following criteria were retrospectively reviewed between November 2019 and July 2020: aged ≥18 years with *EGFR* mutation-positive, TKI-naïve, advanced NSCLC harboring uncommon *EGFR* mutations; and treated with either afatinib, gefitinib, erlotinib, or osimertinib in the first- or second-line setting within regular clinical practice. *EGFR* mutation detection was undertaken locally using different methodologies as per standard care. Information on methodology used and source of material (biopsy, cytology, and blood) was collected.

Patients receiving each agent were categorized hierarchically according to tumor mutation as follows: (1) de novo T790M-positive; (2) exon 20 insertion-positive (but T790M-negative); (3) “major” uncommon mutations (G719X, L861Q, and S768I, with or without any other uncommon mutation except T790M and/or an exon 20 insertion); (4) “other” uncommon mutations (T790M-, exon 20 insertion- and major uncommon mutation-negative). Patients were also categorized according to whether a compound mutation was present, defined as cases where at least 2 *EGFR* mutations were present and at least 1 was an uncommon mutation. Patients must have started EGFR TKI treatment at least 12 months prior to data entry, to avoid early censored data, but did not need to still be on treatment. Patients were excluded if they were treated with an EGFR TKI within a clinical trial or had active brain metastases at the start of EGFR TKI therapy. Patients treated with osimertinib were excluded if they had no further uncommon mutation than acquired T790M on treatment with a first- or second-generation EGFR TKI, as ample clinical evidence already exists of the activity of osimertinib in these patients. A maximum of 15 patients were included per site.

The study was undertaken in compliance with the principles laid down in the Declaration of Helsinki, in accordance with the International Conference on Harmonisation (ICH) Harmonized Tripartite Guideline for Good Clinical Practice, Good Epidemiological Practice and Good Pharmacoepidemiology Practice, and relevant local regulations. Informed and privacy consent signatures were obtained depending on local regulations.

### Outcomes and Assessments

The primary outcome was time-to-treatment failure (TTF) defined as the time from the first dose to the last dose of the EGFR TKI, or death by any cause. Secondary objectives were OS, time on treatment until failure of second-line treatment, overall response rate (ORR) as reported by the investigator, and description of methodology and the material (liquid vs tissue) used for mutation detection.

### Statistical Analysis

A sample size of at least 200 patients was planned, driven by feasibility. It was expected that this would result in approximately 90 patients with major uncommon mutations, 60 patients with exon 20 insertions, and 25 patients with compound mutations. Due to high interest from participating sites, the planned sample size was increased to 250 after a protocol amendment. Time on treatment and OS were estimated using the Kaplan–Meier method. Medians and 2-sided 95% confidence intervals (CIs) were calculated using Greenwood’s variance estimate. For patients still on treatment, TTF was censored at the date of data collection. Comparison of different TKIs and other subgroup analyses were limited to descriptive statistics.

## Results

### Patients

Between December 17 2019 and July 23 2020, a total of 255 patients were included across 36 sites in nine countries and 246 were eligible for analysis (Supplementary Table S1). Of the 9 patients who were ineligible, 3 did not harbor an uncommon *EGFR* mutation, 3 received an EGFR TKI in a clinical trial, 2 had active brain metastases, and 1 had not started EGFR TKI treatment at least 12 months prior to data entry. Most patients with known ethnicity were Asian (90.0%). Patient characteristics were generally similar regardless of which EGFR TKI was received as index therapy (first EGFR-TKI administered; Table 1). Median time from diagnosis to initiation of index therapy was 0.7 months (interquartile range [IQR], 0.4-1.7). Overall, the most common index therapy was afatinib (Table 2). Only 7 patients received osimertinib as index therapy. Most patients (*n* = 226; 91.9%) received an EGFR TKI as first-line treatment (Table 2). Only 21 (8.5%) patients received first-line chemotherapy prior to index EGFR TKI therapy; 9 of these patients had an exon 20 insertion. Most patients (*n* = 140; 56.9%) received more than one line of therapy. The most common second-line treatment was chemotherapy (60.0%) followed by osimertinib (15.0%; Table 2). In total, 31 patients received sequential EGFR TKIs as first- and second-line therapy; 18 received second-line osimertinib, 11 of whom had a documented acquired T790M mutation.

**Table 1.** Baseline characteristics.

	All patients (*n* = 246^a^)	First-generation EGFR TKIs (*n* = 106^b^)	Afatinib (*n* = 132)	Osimertinib (*n* = 7)
Median age, years (range)	69.5 (27-93)	70.5 (42-91)	68.5 (27-93)	71 (56-85)
Female, *n* (%)	138 (56.1)	66 (62.3)	67 (50.8)	5 (71.4)
Smoking status, *n* (%)				
Never	129 (52.4)	64 (60.4)	62 (47.0)	3 (42.9)
Previous	77 (31.3)	24 (22.6)	50 (37.9)	2 (28.6)
Current	25 (10.2)	9 (8.5)	14 (10.6)	2 (28.6)
Unknown	15 (6.1)	9 (8.5)	6 (4.5)	0
Ethnicity, *n* (%)				
Caucasian	23 (9.3)	8 (7.5)	13 (9.8)	1 (14.3)
Asian	206 (83.7)	87 (82.1)	114 (86.4)	5 (71.4)
Unknown/Not collected	17 (6.9)	11 (10.4)	5 (3.8)	1 (14.3)
Stage, *n* (%)				
IIIb/c	37 (15.0)	14 (13.2)	22 (16.7)	0
IV	209 (85.0)	92 (86.8)	110 (83.3)	7 (100)
Histology, *n* (%)				
Adenocarcinoma	239 (97.2)	102 (96.2)	129 (97.7)	7 (100)
Squamous	3 (1.2)	2 (1.9)	1 (0.8)	0
Large cell	2 (0.8)	1 (0.9)	1 (0.8)	0
Other	2 (0.8)	1 (0.9)	1 (0.8)	0
Metastases, *n* (%)				
None	31 (12.6)	16 (15.1)	13 (9.8)	1 (14.3)
Adrenal	15 (6.1)	9 (8.5)	6 (4.5)	0
Bones	82 (33.3)	37 (34.9)	44 (33.3)	1 (14.3)
Brain	17 (6.9)	5 (4.7)	12 (9.1)	0
Liver	22 (8.9)	9 (8.5)	13 (9.8)	0
Lung contralateral	61 (24.8)	22 (20.8)	36 (27.3)	3 (42.9)
Lung ipsilateral	48 (19.5)	17 (16.0)	30 (22.7)	1 (14.3)
Lymph nodes	36 (14.6)	18 (17.0)	16 (12.1)	2 (28.6)
Pleura	57 (23.2)	26 (24.5)	29 (22.0)	2 (28.6)
Spine	9 (3.7)	3 (2.8)	6 (4.5)	0
Other	29 (11.8)	9 (8.5)	17 (12.9)	3 (42.9)
Unknown	7 (2.8)	3 (2.8)	4 (3.0)	0
ECOG PS, *n* (%)				
0	36 (14.6)	9 (8.5)	25 (18.9)	1 (14.3)
1	125 (50.8)	61 (57.5)	59 (44.7)	5 (71.4)
≥2	31 (12.6)	14 (13.2)	17 (12.9)	0
Unknown	54 (22.0)	22 (20.8)	31 (23.5)	1 (14.3)
Treatment lines, *n* (%)				
1	106 (43.1)	35 (33.0)	69 (52.3)	2 (28.6)
2	85 (34.6)	45 (42.5)	36 (27.3)	3 (42.9)
3	30 (12.2)	13 (12.3)	16 (12.1)	1 (14.3)
4	10 (4.1)	4 (3.8)	6 (4.5)	0
5	5 (2.0)	2 (1.9)	3 (2.3)	0
6	4 (1.6)	3 (2.8)	1 (0.8)	0
7	3 (1.2)	2 (1.9)	1 (0.8)	0
8	3 (1.2)	2 (1.9)	0	1 (14.3)

Includes one patient who received both afatinib and gefitinib in the first line and was not allocated to a subgroup.

Includes one patient treated with both erlotinib and gefitinib.

BMI, body mass index; ECOG PS, Eastern Cooperative Oncology Group performance status; EGFR, epidermal growth factor receptor; TKI, tyrosine kinase inhibitor.

**Table 2.** Treatment received.

	Index therapy, *n* (%)	First-line therapy, *n* (%)	Second-line therapy, *n* (%)
EGFR TKI^a^	246 (100.0)	226 (91.9)	52 (37.1)
Afatinib	132 (53.7)	126 (55.8)	11 (21.2)
Gefitinib	70 (28.5)	65 (28.8)	6 (11.5)
Erlotinib	35 (14.2)	28 (12.4)	14 (26.9)
Osimertinib	7 (2.8)	5 (2.2)	21 (40.4)
Afatinib/gefitinib	1 (0.4)	1 (0.4)	0
Gefitinib/erlotinib	1 (0.4)	1 (0.4)	0
Chemotherapy^b^	—	21 (8.5)	84 (60.0)
Chemo-immunotherapy	—	—	4 (2.9)

One patient was treated with gefitinib plus chemotherapy, 27 patients received additional radiotherapy, 1 patient denosumab.

Three patients received chemotherapy + bevacizumab, 12 patients received additional radiation.

EGFR, epidermal growth factor receptor; TKI, tyrosine kinase inhibitor.

In most cases (86.2%), *EGFR* mutational analysis was undertaken on tissue biopsies. Thirteen percent of tests were undertaken on cytology samples and only 1.2% of tests were undertaken on blood samples (Supplementary Table S2). Of re-biopsies undertaken after first-line treatment, 64.7% were undertaken on tissue, 23.5% with blood samples and 8.8% with cytology samples. Of initial mutation tests undertaken prior to first-line treatment, most samples (63.0%) were analyzed with polymerase chain reaction (PCR)-based methodologies. Non-NGS sequencing approaches were used in 15.9% of cases and NGS was used in 8.1% of cases (Supplementary Table S2). As PCR-based detection kits are allele-specific, “other” uncommon mutations, as expected, were most often (61.9%) detected using sequencing methodologies. Of subsequent mutation tests undertaken prior to second-line treatment, 67.6%, 5.9%, and 8.8% were undertaken via PCR-, non-NGS- and NGS-based methodologies, respectively.

Table 3 shows mutation status at the start of index therapy. Patients were categorized into 4 groups: major uncommon mutation (72.8%), exon 20 insertion (11.8%), other uncommon mutation (8.5%), and T790M (6.9%). A third of patients had tumors harboring compound mutations. In some cases, full details of *EGFR* mutations were not provided on pathology reports. For example, full details of the precise nature of exon 20 insertions were only provided in 28.6% of cases (ie, the reports often just stated “exon 20 insertion”). In the “other” *EGFR* mutations category, precise details were only available in 66.7% of cases (eg, some reports just stated “exon 18 deletion”). In all mutation subgroups, afatinib was the most commonly administered index EGFR TKI (“other”: 42.9%; major uncommon: 52.5%; compound: 56.1%; exon 20 insertion: 62.1%; T790M: 64.7%). Baseline and patient characteristics were generally similar across mutation subtypes (Supplementary Table 3). Patients with ECOG PS ≥2 were over-represented in the exon 20 insertion (17.2%) and “other” groups (23.8%; Supplementary Table 3).

**Table 3.** Mutation status at start of first-line treatment.

Mutation category, *n* (%)	All patients (*n* = 246)^c^	First-generation EGFR TKIs (*n* = 106)	Afatinib (*n* = 132)	Osimertinib (*n* = 7)
Major uncommon	179 (72.8)	80 (75.5)	94 (71.2)	4 (57.1)
G719X	112 (45.5)	46 (43.4)	62 (47.0)	4 (57.1)
L861Q	70 (28.5)	34 (32.1)	34 (25.8)	1 (14.3)
S768I	27 (11.0)	6 (5.7)	19 (14.4)	2 (28.6)
Exon 20 insertion^a^	28 (11.4)	9 (8.5)	18 (13.6)	1 (14.3)
T790M	17 (6.9)	4 (3.8)	11 (8.3)	2 (28.6)
Other^b^	21 (8.5)	12 (11.3)	9 (6.8)	0
Compound	81 (32.9)	31 (29.2)	46 (34.8)	4 (57.1)

Unknown (*n* = 6), A763_Y764insFQEA (*n* = 2) A767_V769dup (*n* = 2), D770_N771insSVD (*n* = 2), S768_D770dup (*n* = 1), V769_D770ins (*n* = 1), incomplete description (*n* = 14).

Exon 18 (*n* = 5), V703L + L707W, P753S + L747_S752del, V742F + A743V + H773R, E709X, K714N, A864P, exon19ins, F712C, K716E, K719A, L747_P753delins, L861R, R776H, S720F, S791I, T710S (all *n* = 1).

One patient who received first-line chemotherapy and second-line erlotinib had unknown *EGFR* mutation status.

EGFR, epidermal growth factor receptor; TKI, tyrosine kinase inhibitor.

### Clinical Outcomes

**Figure 1.**
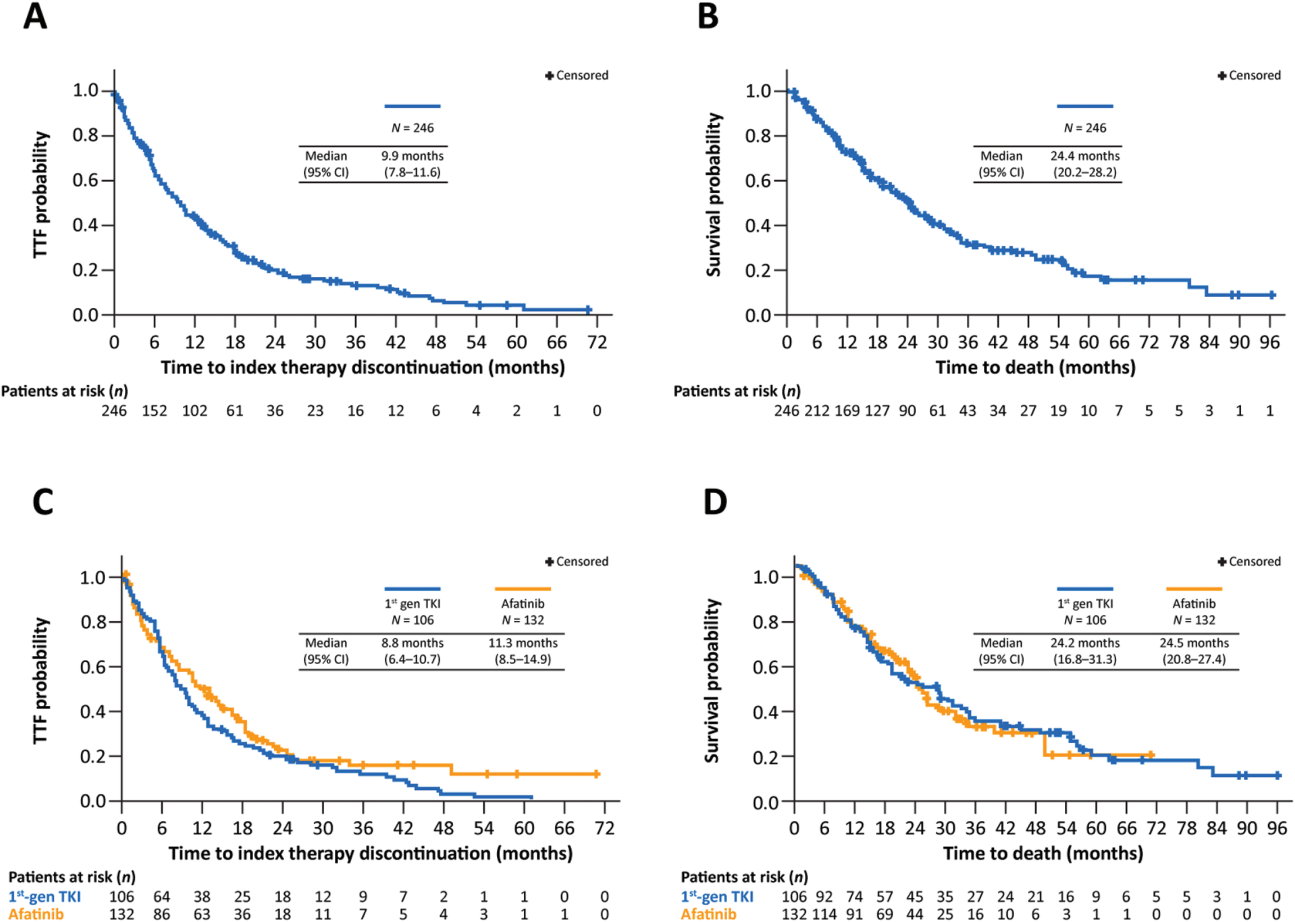
Time-to-treatment failure **(A)** and OS **(B)** in the overall uncommon mutation dataset (*n* = 246). Time-to-treatment failure **(C)** and OS **(D)** according to whether patients received a first-generation EGFR TKI (*n* = 106) or afatinib (*n* = 132).

After a median follow-up of 19.1 months (IQR, 10.5-31.3), median TTF with the index EGFR TKI was 9.9 months (95% CI, 7.8-11.6; Fig. 1A, Table 4) and median OS was 24.4 months (95% CI, 20.2-28.2; Fig. 1B, Table 4). The ORR was 43.4% and median duration of response was 10.0 months (IQR, 5.0-16.0; Table 5). In patients receiving the index EGFR TKI as first-line treatment, median TTF was 10.5 months (95% CI, 8.5-12.6) and ORR was 45.3%. When EGFR TKIs were received in a second-line setting (*n* = 20), median TTF was 5.8 months (IQR, 2.6-12.8). These 20 patients were treated with first-line chemotherapy, with a median TTF of 6.6 months (95% CI, 4.4-7.9), ORR of 41.2% and duration of response of 4.0 months (IQR, 3.0-7.0). Further exploratory analysis was undertaken on outcomes for first- and second-generation EGFR TKIs. Median TTF with first-generation EGFR TKIs and afatinib was 8.8 months (95% CI, 6.4-10.7) and 11.3 months (95% CI, 8.5-14.9), respectively (Fig. 1C, Table 4). Median OS was 24.2 months (95% CI, 16.8-31.3) and 24.5 months (95% CI, 20.8-27.4), respectively (Fig. 1D, Table 4). Outcomes with osimertinib were not assessed due to small sample size.

**Table 4.** TTF and OS in patient subgroups.

Median time-to-treatment failure, months (95% CI)						
	Any TKI *n* = 246		First-generation TKIs *n* = 106		Afatinib *n* = 132	
All patients	9.9 (7.8-11.6)		8.8 (6.4-10.7)		11.3 (8.5-14.9)	
Mutation category						
Major uncommon	*n* = 179	11.3 (9.2-14.3)	*n* = 80	9.8 (7.6-12.9)	*n* = 94	14.3 (10.5-17.8)
Compound	*n* = 82	12.3 (8.5-15.5)	*n* = 32	12.4 (7.4-27.9)	*n* = 46	12.6 (6.9-15.7)
Others	*n* = 21	7.4 (2.1-12.8)	*n* = 12	7.3 (0.6-12.6)	*n* = 9	10.8 (0.2-17.9)
Exon 20 insertion	*n* = 29	5.5 (2.9-10.6)	*n* = 10	5.2 (1.4-9.6)	*n* = 18	8.3 (3.1-18.5)
T790M	*n* = 17	2.8 (2.1-7.4)	*n* = 4	2.1 (0.9–2.3)	*n* = 11	5.7 (1.9-12.6)
Baseline brain metastases (major uncommon)						
No (*n*= 162)	10.7 (9.1-14.2)		—		—	
Yes (*n*= 17)	17.3 (7.7-24.5)		—		—	
ECOG PS (major uncommon)						
<2 (*n* = 122)	11.5 (8.5-14.7)		—		—	
≥2 (*n* = 20)	8.6 (4.6-15.7)		—		—	
Overall survival, months (95% CI)						
All patients	24.4 (20.2-28.2)		24.2 (16.8-31.3)		24.5 (20.8-27.4)	
Mutation category						
Major uncommon	*n* = 179	25.7 (19.7-30.2)	*n* = 80	28.5 (18.6-34.7)	*n* = 94	24.5 (18.4-28.6)
Compound	*n* = 82	28.7 (22.5-33.0)	*n* = 32	31.3 (15.6-80.1)	*n* = 46	23.4 (16.0-34.5)
Others	*n* = 21	13.4 (5.9–24.8)	*n* = 12	12.8 (3.7-55.8)	*n* = 9	20.2 (0.3-24.8)
Exon 20 insertion	*n* = 29	22.5 (14.3-49.7)	*n* = 10	21.0 (1.7-62.4)	*n* = 18	22.5 (9.9–NR)
T790M	*n* = 17	32.7 (11.1-83.2)	*n* = 4	14.2 (11.1-83.2)	*n* = 11	NR (10.3–NR)
Baseline brain metastases (major uncommon)						
No (*n* = 162)	25.7 (19.4-30.2)		—		—	
Yes (*n* = 17)	33.9 (11.5-49.6)		—		—	
ECOG PS (major uncommon)						
<2 (*n* = 122)	28.5 (19.7-34.5)		—		—	
≥2 (*n* = 20)	14.3 (9.1-23.4)		—		—	

CI, confidence interval; ECOG PS, Eastern Cooperative Oncology Group performance status; NR, not reported.

**Table 5.** Response rates and duration of response to index EGFR TKI treatment (evaluable patients).

	Any TKI *N* = 221			First-generation TKIs *N* = 93			Afatinib *N* = 121			Osimertinib *N* = 6		
	ORR, %		DoR, mos (IQR)	ORR, %		DoR, mos (IQR)	ORR, %		DoR, mos (IQR)	ORR, %		DoR, mos
All patients	43.4		10 (5-16)	44.1		6 (3-12)	43.8		12 (5.5-17)	16.7		11
Major uncommon	*n* = 167	49.1	10 (4.5-17)	*n* = 74	47.3	6.5 (2.5-11.5)	*n* = 89	50.6	12 (7-17)	*n* = 3	33.3	11
Compound mutation	*n* = 72	48.6	10 (3-16)	*n* = 29	48.3	6 (2-24)	*n* = 40	52.5	10 (5-16)	*n* = 3	0	—
Others	*n* = 16	43.8	7.5 (4.5-10.5)	*n* = 9	55.6	4.5 (3-6)	*n* = 7	28.6	10.5 (9-12)	*n* = 0	—	—
Exon 20 insertion	*n* = 23	17.4	19.3 (5.5-33)	*n* = 6	16.7	33	*n* = 16	18.8	5.5	*n* = 1	0	—
T790M	*n* = 15	20.0	6 (2-12)	*n* = 4	0	—	*n* = 9	33.3	6 (2-12)	*n* = 2	0	—

EGFR, epidermal growth factor receptor; DoR, duration of response; IQR, interquartile range; mos, months; ORR, objective response rate; TKI, tyrosine kinase inhibitor.

**Figure 2.**
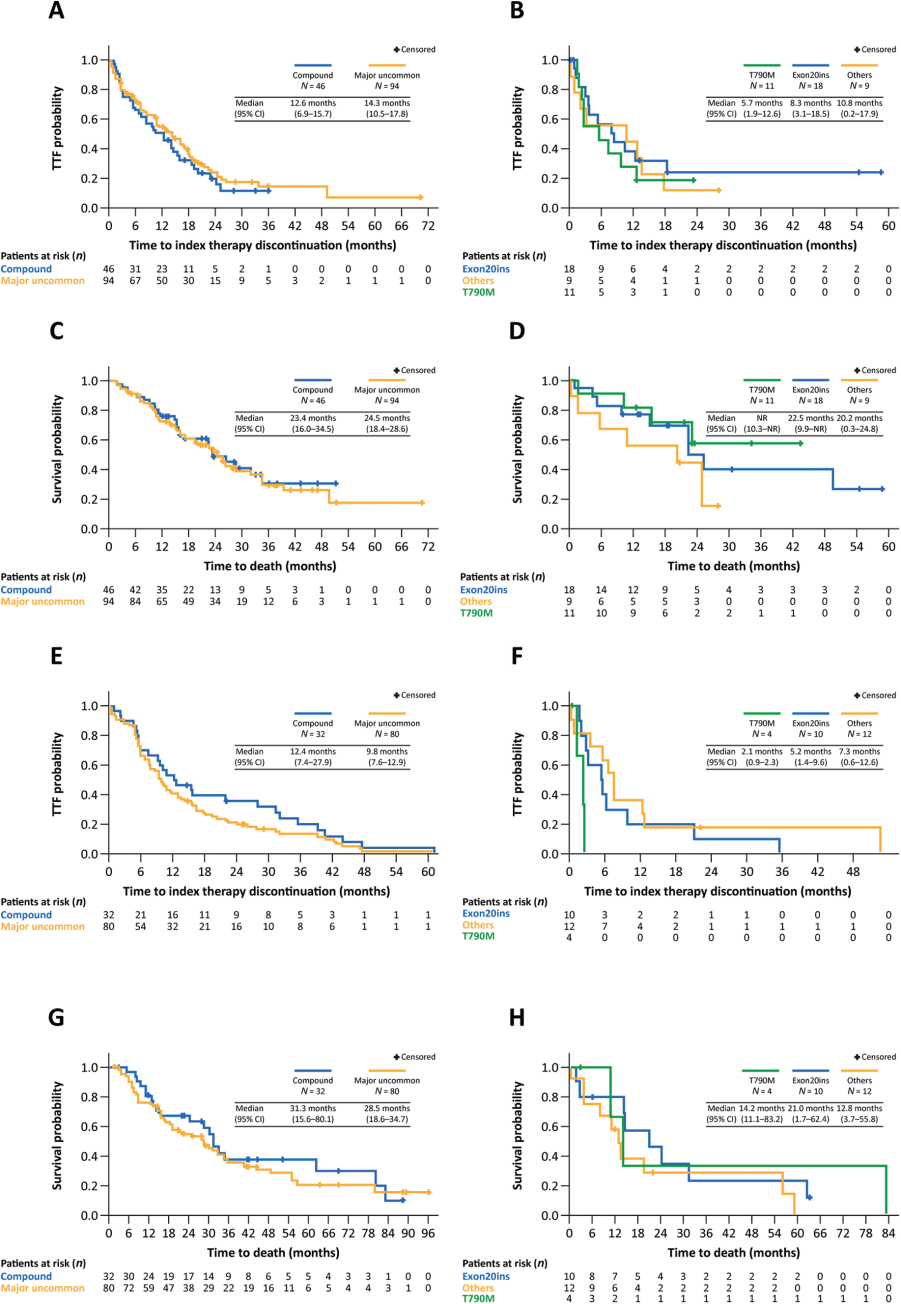
Time-to-treatment failure **(A, B)** and OS **(C, D)** in patients who received afatinib according to uncommon mutation category. Time-to-treatment failure **(E, F)** and OS **(G, H)** in patients who received a first-generation EGFR TKI according to uncommon mutation category.

Table 4 summarizes TTF and OS with index EGFR TKIs according to uncommon mutation category. As expected, outcomes varied according to category, with the best results seen in patients with major uncommon and compound mutations. With afatinib, median TTF ranged from 5.7 months in the T790M group to 14.3 months in the major uncommon mutation group (Fig. 2A, B). Median TTF was 12.6 months in the compound mutation group. Of note, however, 30.4% of the compound mutation group had a constituent T790M or exon 20 insertion mutation (Supplementary Table S4). Median OS was approximately 2 years in most mutation categories (Fig. 2C, D). With first-generation EGFR TKIs, median TTF ranged from 2.1 months in the T790M group to 12.4 months in the compound mutation group, of which 21.9% had a constituent T790M or exon 20 insertion mutation (Fig. 2E, F; Supplementary Table S4). Median OS ranged from 14.2 months in the T790M group to 31.3 months in the compound mutation group (Fig. 2G, H). Table 5 shows ORRs and duration of response according to mutation category and EGFR TKI received. The ORR with afatinib was 43.8% (median duration of response: 12.0 months) and the ORR with first-generation EGFR TKIs was 44.1% (median duration of response: 6.0 months). Objective response rates were highest in the major uncommon and compound mutation groups. However, there was also notable activity in the “other” category. Nearly a fifth of the exon 20 insertion group eligible for response evaluation responded to treatment with EGFR TKIs. Full details of the nature of the exon 20 insertion were available for only 2 of the responding patients (A763_T764insFQEA and M766_A767insASV, both of whom responded to afatinib).

In this study, most patients (76.8%) received the approved starting dose of EGFR TKIs (gefitinib: 250 mg; erlotinib 150 mg; afatinib 40 mg). Time-to-treatment failure, OS, and ORR outcomes in these patients are shown in Supplementary Tables 5 and 6 and Supplementary Fig. S1. Time-to-treatment failure and OS according to mutation subtype in patients who were treated with afatinib according to label (first-line, recommended starting dose) are shown in Supplementary Fig. 2.

## Discussion

This retrospective study provides valuable insights into how patients with *EGFR* mutation-positive NSCLC are treated in everyday clinical practice if an uncommon *EGFR* tumor mutation is detected. Our study suggests that EGFR TKIs may be widely regarded as standard of care in this setting, with over 90% of patients receiving a first-line EGFR TKI. Median time from NSCLC diagnoses to index therapy was less than 1 month. While the study was not designed to compare the activity of different treatment regimens, EGFR TKIs demonstrated encouraging efficacy. In the overall dataset, independent of mutation category or type of EGFR TKI administered, median TTF was close to 10 months, OS was more than 2 years and ORR was 43%. These findings are particularly promising given the “real-world” setting: the median age was nearly 70 years old, 16% of patients had an ECOG PS of ≥2 and 7% had brain metastases at diagnosis.

The most commonly used EGFR TKI in this study was afatinib, with few patients receiving osimertinib. This observation possibly reflects the current availability of more clinical data supporting the use of afatinib against uncommon mutations compared with other EGFR TKIs at this time, coupled with the fact that osimertinib has been widely available for less time than afatinib. Based on sub-analysis of the prospective LUX-Lung 2, 3, and 6 trials^22^ (which included patients with uncommon mutations), the US Food and Drug Administration (FDA) and the European Medicines Agency approved afatinib for NSCLC patients with any sensitizing *EGFR* mutation (in addition to Del19 and L858R mutations).^39^ Few patients received first-line chemotherapy (mainly those with exon 20 insertions), but the median TTF of 6.6 months was shorter than that observed with EGFR TKIs. While over 40% of patients responded to chemotherapy, the median duration of response was only 4 months. Our findings therefore indicate that first-line TKIs should be considered for the treatment of NSCLC with uncommon *EGFR* mutations in order to delay/avoid use of chemotherapy in this setting. Indeed, the observation that most patients received at least 2 lines of therapy, and ECOG PS remained largely stable following first-line EGFR TKI treatment suggest that the use of first-line EGFR TKIs in this setting does not negatively impact on patients’ general well-being, thus facilitating sequential treatment strategies.

This study further illustrates that uncommon *EGFR* mutations are highly heterogeneous and compound mutations are common in patients with *EGFR* mutation-positive NSCLC. However, in this community setting, allele-specific PCR-based methodologies were by far the most commonly used technique to detect *EGFR* mutations. Broader NGS techniques were not routinely used and molecular reports were not always sufficiently detailed. Also, blood-based *EGFR* mutation detection techniques, which could broaden opportunities to test for *EGFR* mutations,^40^ were not widely used. Overall, therefore, it seems that mutation detection methodologies in “real-world” practice could be improved in order to increase the frequency and breadth of uncommon activating *EGFR* mutations detected.

As expected, the activity of EGFR TKIs in this study varied according to uncommon mutation category. Consistent with previous preclinical and clinical findings,^22,23,26^ afatinib was active against major uncommon mutations, with an ORR of 51% and TTF of 14.3 months. Although first-generation EGFR TKIs have shown variable activity against major uncommon mutations in previous studies,^38^ they also demonstrated robust activity in this study with an ORR of 51% and median TTF of 9.8 months. Overall, therefore, our data provide further evidence that patients with a G719X, L861Q, and S768I should receive an EGFR TKI as first-line treatment of choice.

To the best of our knowledge, this is the first study to assess OS specifically in a cohort of patients with tumors harboring uncommon *EGFR* mutations treated with EGFR TKIs. Median OS was similar with afatinib and first-generation TKIs and was over 2 years in both cases. The OS in the 2 groups was likely confounded by the high uptake of subsequent treatments and less censoring for the first-generation EGFR TKIs due to their longer availability. Ultimately, however, it appears that OS seems shorter than that observed for common *EGFR* mutations. Compound mutations were also sensitive to both afatinib and first-generation EGFR TKIs in this study with ORRs of around 50% and median TTF of more than a year. Again, these findings are consistent with previous analyses, although sensitivity appears to be largely driven by the nature of concomitant mutations.^38^ Median OS was 23.4 and 31.3 months with afatinib and first-generation EGFR TKIs, respectively, in patients with compound mutations. Of note, a higher proportion of compound mutations contained T790M or an exon 20 insertion in the afatinib group than the first-generation EGFR TKI group, which will likely have influenced activity.

While the major uncommon and compound mutation categories were the most sensitive to EGFR TKIs, notable activity was also observed in the “other” and exon 20 insertion categories. As discussed, these categories are highly heterogeneous and it is important that the precise nature of the mutations is defined. Given the rarity of individual variants, there is a paucity of clinical data to help drive appropriate treatment decisions. Nevertheless, a number of online databases of clinical cases have been developed to help physicians when considering treatment options for tumors with very rare *EGFR* variants eg, the afatinib uncommon mutations database (www.uncommonEGFRmutations.com), MyCancerGenome (www.mycancergenome.org) and OncoKB (www.oncokb.org). Consistent with previous observations, modest activity was seen against exon 20 insertions. Although a number of novel agents, including poziotinib, mobocertinib, and amivantamab, are undergoing clinical development in this setting,^41^ it remains an area of unmet need. While exon 20 insertions are generally considered insensitive to EGFR TKIs, preclinical analysis indicates that certain variants are sensitive, especially to second- and third-generation EGFR TKIs.^23,28,42^ Indeed, case study reports and small cohort studies have demonstrated durable responses against exon 20 insertions such as A763_Y764insFQEA, A767delinsASVD, A767_Y768insSVA, and A767_V769dup.^43-46^ These observations further emphasize the necessity for precise molecular definition of mutations, and further collection of clinical data. Finally, as expected, activity of both first-generation TKIs and afatinib against T790M was poor. While median OS was 14.2 and 32.7 months, respectively, this probably reflects the impact of subsequent therapies. As both first- and second-generation EGFR TKIs are widely accepted to have low activity in this setting, osimertinib is the clear treatment of choice for T790M.

Given our retrospective analysis of medical and electronic health records, this study had several limitations. Clearly there was potential for selection bias, as patients had to have a documented uncommon mutation and must have received an EGFR TKI at some point in their treatment history in order to be included. Therefore, the results are not generalizable to all patients with tumors harboring uncommon *EGFR* mutations. Despite this inherent limitation, efforts were undertaken to minimize the potential for selection bias. For instance, to avoid differential center influence on study results, a maximum of 15 consecutive patients per site were included. Furthermore, patients must have initiated EGFR TKI treatment at least 12 months prior to data entry to avoid early censoring. Patients treated with the index therapy in the second line might have impacted the outcomes (especially OS). However, numbers were too small (*n* = 20) to analyze them separately. The analyses of activity according to the EGFR TKI received did not include formal testing for statistical significance. Moreover, few patients received osimertinib so it was not possible to undertake any meaningful analysis regarding the activity of this agent against uncommon *EGFR* mutations. Also, as the study was reliant on analysis of electronic case report forms, data were often incomplete, particularly regarding the precise *EGFR* genotype. Finally, although we categorized *EGFR* mutations, the “other”, exon 20 insertions, and compound mutation groups remained highly heterogeneous thus complicating the interpretation of clinical outcomes data.

In conclusion, this retrospective study, undertaken in a real-world setting, demonstrated the frequency and diversity of uncommon *EGFR* mutations. Also, while selection bias cannot be discounted, the data suggest that EGFR TKIs may be the preferred first-line treatment option in patients with tumors harboring such mutations in “real-world” clinical practice. Strongest outcomes were observed in the major uncommon and compound mutation categories. Our findings provide further evidence that treatment with an EGFR TKI should be considered as standard of care for most patients with uncommon mutations. However, optimal patient management in real-world practice requires improvements in pathology reports and greater implementation of NGS methodology.

## Supplementary Material

oyac022_suppl_Supplementary_Material

## Data Availability

The datasets generated and analyzed during the study are available from A.M. on reasonable request.
