# Supplementary material for: Tyrosine Kinase Inhibitor Activity in Patients with NSCLC Harboring Uncommon EGFR Mutations: A Retrospective International Cohort Study (UpSwinG)
Source: Oncologist. 2022 Mar 11;27(4):255–65. doi: 10.1093/oncolo/oyac022 (PMC8982383; doi:10.1093/oncolo/oyac022)

**Supplementary Table 1.** Details of enrolment across study sites

|  | **Total number of enrolled patients** | | **Total number of eligible patients** | |
| --- | --- | --- | --- | --- |
|  | ***N*** | **%** | ***N*** | **%** |
| **Overall** | **255** | **100.0** | **246** | **96.5** |
| KR-02 — Samsung Medical Center (Seoul) — Prof. Jung | 15 | 5.9 | 15 | 6.1 |
| TW-01 — National Taiwan University Hospital (Taipei) — Dr. Shih | 15 | 5.9 | 15 | 6.1 |
| TW-02 — China Medical University Hospital (Taichung) — Dr. Hsia | 15 | 5.9 | 15 | 6.1 |
| TW-03 — Kaohsiung Medical University Chung-Ho Memorial Hospital (Kaohsiung) — Dr. Hung | 15 | 5.9 | 15 | 6.1 |
| TW-06 — Taichung Veterans General Hospital (Taichung) — Dr. Yang | 15 | 5.9 | 15 | 6.1 |
| KR-05 — Chonnam National University Hwasun Hospital (Hwasun-gun) —  Prof. Kim | 14 | 5.5 | 14 | 5.7 |
| KR-09 — Kyunghee University Medical Center (Seoul) — Prof. Lee | 14 | 5.5 | 14 | 5.7 |
| JP-02 — NHO Kyushu Cancer Center (Fukuoka-shi) — Dr. Okamoto | 11 | 4.3 | 11 | 4.5 |
| JP-03 — Osaka International Cancer Institute (Osaka-shi) — Dr. Kumagai | 10 | 3.9 | 7 | 2.8 |
| KR-06 — Gachon University Gil Medical Center (Incheon) — Prof. Ahn | 10 | 3.9 | 9 | 3.7 |
| JP-01 — Niigata Cancer Center Hospital (Niigata-shi) — Dr. Miura | 9 | 3.5 | 8 | 3.3 |
| JP-11 — Kobe City Hospital Organization Kobe City Medical Center General Hospital (Kobe-shi) — Dr. Sato | 9 | 3.5 | 9 | 3.7 |
| KR-03 — Chonbuk National University Hospital (Jeonju-si) — Prof. Lee | 9 | 3.5 | 9 | 3.7 |
| KR-04 — Pusan National University Hospital (Busan) — Prof. Lee | 9 | 3.5 | 7 | 2.8 |
| KR-07 — Inje University Haeundae Paik Hospital (Busan) — Prof. Lee | 9 | 3.5 | 8 | 3.3 |
| FR-03 — CHU Strasbourg - Nouvel Hôpital Civil (Strasbourg) — Prof. Mascaux | 7 | 2.7 | 7 | 2.8 |
| KR-08 — Asan Medical Center (Seoul) — Prof. Kim | 7 | 2.7 | 7 | 2.8 |
| GB-02 — Royal Cornwall Hospital (Truro) — Dr. Stewart | 6 | 2.4 | 6 | 2.4 |
| KR-12 — Severance Hospital, Yonsei University Health System (Seoul) —  Prof. Lim | 6 | 2.4 | 6 | 2.4 |
| FR-01 — Centre Hospitalier Intercommunal de Créteil (Creteil Cedex) —  Dr. Auliac | 5 | 2.0 | 5 | 2.0 |
| AT-01 — Klinik Floridsdorf (Vienna) — Dr. Hochmair | 4 | 1.6 | 4 | 1.6 |
| IT-09 - Istituto Clinico Humanitas (Rozzano) — Dr. Rossi | 4 | 1.6 | 4 | 1.6 |
| JP-05 — NHO Yamaguchi - Ube Medical Center (Ube-shi) — Dr. Aoe | 4 | 1.6 | 4 | 1.6 |
| TW-04 — Taipei Veterans General Hospital (Taipei) — Dr. Chen | 4 | 1.6 | 4 | 1.6 |
| ES-03 — Hospital Universitario Fundacion Alcorcon (Alcorcon) —  Dr. Mielgo Rubio | 3 | 1.2 | 3 | 1.2 |
| IT-05 — Azienda Ospedaliera Universitaria Careggi (Firenze) — Dr. Scotti | 3 | 1.2 | 3 | 1.2 |
| JP-09 — Kanagawa Cancer Center (Kawasaki-shi) — Dr. Saito | 3 | 1.2 | 3 | 1.2 |
| KR-01 — Ajou University Hospital (Suwon) — Prof. Lee | 3 | 1.2 | 3 | 1.2 |
| KR-10 — Chungnam National University Hospital (Daejeon) — Prof. Lee | 3 | 1.2 | 3 | 1.2 |
| TW-05 — Kaohsiung Chang Gung Memorial Hospital (Kaohsiung) — Dr. Chang | 3 | 1.2 | 3 | 1.2 |
| AT-02 — Kepler University Hospital (Linz) — Prof. Lamprecht | 2 | 0.8 | 2 | 0.8 |
| DE-02 — Gemeinschaftskrankenhaus Havelhohe (Berlin) — Dr. Matthes | 2 | 0.8 | 2 | 0.8 |
| ES-01 — ICO Badalona - Hospital Universitari Germans Trias i Pujol (Badalona) — Dr. Moran | 2 | 0.8 | 2 | 0.8 |
| IT-03 — Istituto Nazionale Tumori Fondazione G. Pascale (Napoli) —  Dr. Morabito | 2 | 0.8 | 1 | 0.4 |
| JP-14 — NHO Himeji Medical Center (Himeji-shi) — Dr. Kawamura | 2 | 0.8 | 2 | 0.8 |
| JP-04 — KKR Kumamoto Chuo Hospital (Kumamoto-shi) — Dr. Ushijima | 1 | 0.4 | 1 | 0.4 |

**Supplementary Table 2.** Approaches for detection of mutations at first-line treatment start

|  | **Eligible patients (*N* = 246)** |
| --- | --- |
| Biologic sample(s) used for mutation testing  Tissue, Histological sample (solid biopsy)  Cytological sample  Blood (liquid biopsy)  Other  Unknown | 212 (86.2%)  32 (13.0%)  3 (1.2%)  2 (0.8%)  3 (1.2%) |
| Methodology used for mutation testing  Amplification Refractory Mutation System (ARMS)  Roche  PCR-based techniques  ddPCR  PCR clamping  Real-time PCR  Massarray Genotyping  Targeted PCR  PCR (COBAS)  PCR-invader assay  PCR (type not specified)  Sequencing  Sanger sequencing  Pyrosequencing  Other  Unknown  NGS  Targeted NGS  Whole-genome sequencing  Unknown  Unknown | 4 (1.6%)  4 (1.6%)  155 (63.0%)  38 (15.4%)  39 (15.9%)  29 (11.8%)  23 (9.3%)  15 (6.1%)  3 (1.2%)  3 (1.2%)  3 (1.2%)  39 (15.9%)  12 (4.9%)  3 (1.2%)  9 (3.7%)  15 (6.1%)  20 (8.1%)  14 (5.7%)  3 (1.2%)  3 (1.2%)  42 (17.1%) |

NGS, next-generation sequencing; PCR, polymerase chain reaction.

**Supplementary Table 3.** Patient baseline and disease characteristics according to uncommon mutation category

| **Characteristics, n (%)** | **T790M *n* = 17** | **Exon 20 insertion *n* = 29** | **Major uncommon* *n* = 179** | **Other *n* = 21** | **Compound^†^ *n* = 82** |
| --- | --- | --- | --- | --- | --- |
| Ethnicity  Asian  Non-Asian  Unknown | 16 (94.1)  0  1 (5.9) | 22 (75.9)  3 (10.3)  4 (13.8( | 156 (87.2)  15 (8.4)  8 (4.5) | 12 (57.1)  5 (23.8)  4 (19.0) | 71 (86.6)  7 (8.5)  4 (4.9) |
| Brain metastases | 1 (5.9) | 1 (3.4) | 20 (11.2) | 0 | 4 (4.9) |
| ECOG ≥2 | 1 (5.9) | 5 (17.2) | 20 (11.2) | 5 (23.8) | 7 (8.5) |
| First-line EGFR-TKI | 16 (94.1) | 20 (69.0) | 169 (94.4) | 21 (100.0) | 77 (93.9) |
| More than one line of therapy | 12 (70.6) | 20 (69.0) | 98 (54.7) | 10 (47.6) | 48 (58.5) |
| Index EGFR TKI therapy  Afatinib  Gefitinib  Erlotinib  Osimertinib | 11 (64.7)  4 (23.5)  0  2 (11.8) | 18 (62.1)  3 (10.3)  7 (24.1)  1 (3.4) | 94 (52.5)  55 (30.7)  24 (13.4)  4 (2.2) | 9 (42.9)  8 (38.1)  4 (19.0)  0 | 46 (56.1)  22 (26.8)  9 (11.0)  4 (4.9) |
| Methodology used for mutation testing at start of index therapy  PCR  Sequencing | 11 (64.7)  1 (5.9) | 12 (41.4)  6 (20.1) | 118 (65.9)  32 (17.9) | 5 (23.8)  13 (61.9) | 56 (68.3)  16 (19.5) |
| Material used for mutation classification at start of index therapy  Tissue  Liquid  Cytology |  |  |  |  |  |
|  | 14 (82.4)  0  2 (11.8) | 19 (65.5)  0  0 | 149 (83.2)  2 (1.1)  23 (12.8) | 17 (81.0)  0  4 (19.0) | 65 (79.3)  0  14 (17.1) |

ECOG PS, Eastern Cooperative Oncology Group performance status; EGFR, epidermal growth factor receptor; PCR, polymerase chain reaction; TKI, tyrosine kinase inhibitor. *One patient received gefitib/erlotinib; ^†^One patient received afatinib/gefitinib

**Supplementary Table 4.** Details of compound mutations

|  | **Common *EGFR* mutation**  **(Del19/L858R)** | **Exon 20 insertion** | **Major uncommon mutation** | **Other** |
| --- | --- | --- | --- | --- |
| Compound mutations treated with afatinib (*n* = 46) | | | | |
| T790M, *n* = 11  Exon 20 insertion, *n* = 3  Major uncommon, *n* = 31  Others, *n* = 1 | 11 (100)  2 (67)  8 (26)  1 (100) | 0  0  0  0 | 0  1 (33)  23 (74)  0 | 0  0  4 (13)  1 (100) |
| Compound mutations treated with first-generation EGFR TKIs (*n* = 32)* | | | | |
| T790M, *n* = 4  Exon 20 insertion, *n* = 3  Major uncommon, *n* = 19  Others, *n* = 6 | 4 (100)  3 (100)  17 (90)  5 (83) | 0  0  0  0 | 0  0  6 (32)  0 | 0  0  4 (21)  1 (17) |
| Compound mutations treated with osimertinib (*n* = 4) | | | | |
| T790M, *n* = 2  Exon 20 insertion, *n* = 0  Major uncommon, *n* = 2  Others, *n* = 0 | 2 (100)  0  0  0 | 0  0  0  0 | 0  0  2 (100)  0 | 0  0  0  0 |

EGFR, epidermal growth factor receptor; TKI, tyrosine kinase inhibitor.

*One patient was treated with afatinib followed by gefitinib in the first line

**Supplementary Table 5.** TTF and OS in patient subgroups who received approved starting doses of EGFR TKIs (gefitinib 250 mg; erlotinib 150 mg; afatinib 40 mg) as first-line treatment

|  | **Any TKI**  ***N* = 189** | | **First-generation TKIs**  ***N* = 91** | | **Afatinib**  ***N* = 93** | |
| --- | --- | --- | --- | --- | --- | --- |
| **Median time to treatment failure, months (95% CI)** | | | | | | |
| All patients | 10.7 (9.2–12.9) | | 9.3 (7.3–12.3) | | 12.8 (10.5–17.8) | |
| Mutation category  Major uncommon  Compound  Others  Exon 20 insertion  T790M | *n* = 144  *n* = 63  *n* = 15  *n* = 14  *n* = 16 | 11.9 (9.3–16.1)  12.6 (7.4–18.8)  11.5 (5.6–13.7)  10.6 (3.7–21.0)  2.8 (1.9–7.4) | *n* = 71  *n* = 26  *n* = 11  *n* = 5  *n* = 4 | 10.4 (7.8–15.4)  14.0 (7.4–31.9)  7.4 (0.6–12.6)  6.0 (1.4–21.0)  2.1 (0.9–2.3) | *n* = 69  *n* = 34  *n* = 4  *n* = 9  *n* = 11 | 15.7 (10.5–18.7)  12.6 (5.6–19.4)  13.3 (10.8–NR)  15.5 (1.9–NR)  5.7 (1.9–12.6) |
| Baseline brain metastases (major uncommon)  No (*n* = 129)  Yes (*n* = 15) | 10.8 (8.6–15.4)  17.4 (7.0–31.9) | | -  - | | -  - | |
| ECOG PS (major uncommon)  <2 (*n* = 97)  ≥2 (*n* = 16) | 12.5 (9.1–16.7)  7.7 (4.6–15.7) | | -  - | | -  - | |
| **Overall survival, months (95% CI)** | | | | | | |
| All Patients | 25.6 (21.4–31.9) | | 28.2 (19.1–34.7) | | 24.8 (20.8–34.0) | |
| Mutation category  Major uncommon  Compound  Others  Exon 20 insertion  T790M | *n* = 144  *n* = 63  *n* = 15  *n* = 14  *n* = 16 | 28.5 (21.4–33.9)  30.2 (22.5–34.8)  20.2 (8.1–55.8)  22.5 (14.5–NR)  23.0 (11.1–83.2) | *n* = 71  *n* = 26  *n* = 11  *n* = 5  *n* = 4 | 30.2 (19.4–35.7)  33.0 (14.2–83.2)  13.4 (3.8–55.8)  21.0 (14.3–NR)  14.2 (11.1–83.2) | *n* = 69  *n* = 34  *n* = 4  *n* = 9  *n* = 11 | 24.5 (18.4–34.0)  23.4 (16.0–34.5)  24.8 (20.2–NR)  22.5 (9.9–NR)  NR (10.3–NR) |
| Baseline brain metastases (major uncommon)  No (*n* = 129)  Yes (*n* = 15) | 28.2 (19.4–33.0)  33.9 (17.0–49.6) | | -  - | | -  - | |
| ECOG PS (major uncommon)  <2 (*n* = 97)  ≥2 (*n* = 16) | 28.8 (20.8–40.8)  16.0 (9.1–23.4) | | -  - | | -  - | |

CI, confidence interval; ECOG PS, Eastern Cooperative Oncology Group performance; EGFR, epidermal growth factor receptor; NR, not reached; TKIs, tyrosine kinase inhibitors.

**Supplementary Table 6.** Response rates and duration of response to index EGFR TKI treatment in patients who received approved starting doses (gefitinib 250 mg; erlotinib 150 mg; afatinib 40 mg; evaluable patients)

|  | **Any TKI**  ***N* = 174** | | | **First-generation TKIs**  ***N* = 84** | | | **Afatinib**  ***N* = 86** | | | |
| --- | --- | --- | --- | --- | --- | --- | --- | --- | --- | --- |
|  | **ORR, %** | | **DoR, mos (IQR)** | **ORR, %** | | **DoR, mos (IQR)** | **ORR, %** | | **DoR, mos (IQR)** |  |
| All patients | 45.4 | | 10 (4–14) | 47.6 | | 6 (3–11) | 44.2 | | 12 (5–17) |  |
| Major uncommon | *n* = 136 | 50.0 | 10 (4–17) | *n* = 68 | 51.5 | 6.5 (2.5–11.5) | *n* = 65 | 49.2 | 12 (5–17) |  |
| Compound mutation | *n* = 56 | 50.0 | 10 (3–12) | *n* = 25 | 52.0 | 4.5 (2–11) | *n* = 29 | 51.7 | 10 (3–17) |  |
| Others | *n* = 12 | 50.0 | 6 (3–12) | *n* = 9 | 55.6 | 4.5 (3–6) | *n* = 3 | 33.3 | 12 |  |
| Exon 20 insertion | *n* = 12 | 16.7 | NR | *n* = 3 | 0 | - | *n* = 9 | 22.2 | - |  |
| T790M | *n* = 14 | 21.4 | 6 (2–12) | *n* = 4 | 0 | - | *n* = 9 | 33.3 | 6 (2–12) |  |

DoR, duration of response; IQR, interquartile range; mos, months; NR, not reached; ORR, objective response rate.

**Supplementary Figure 1.** TTF **(A)** and OS **(B)** in the overall uncommon mutation dataset in patients who received approved starting EGFR TKI doses (gefitinib 250 mg; erlotinib 150 mg; afatinib 40 mg; *n* = 189). TTF **(C)** and OS **(D)** according to whether patients received the approved dose of a first-generation EGFR TKI or afatinib.


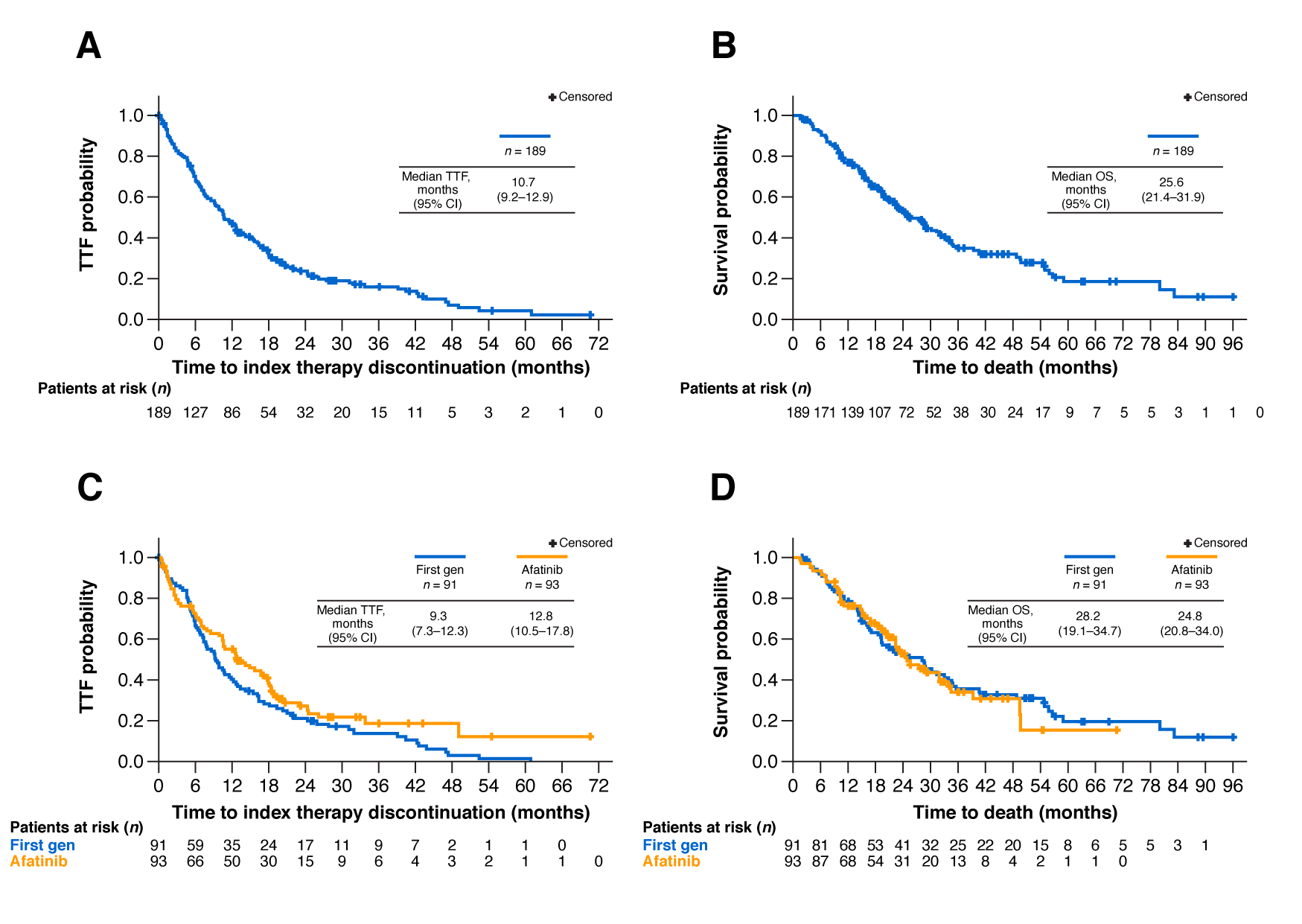


**Supplementary Figure 2.** TTF **(A, B)** and OS **(C, D)** according to uncommon mutation category in patients who received afatinib according to label.


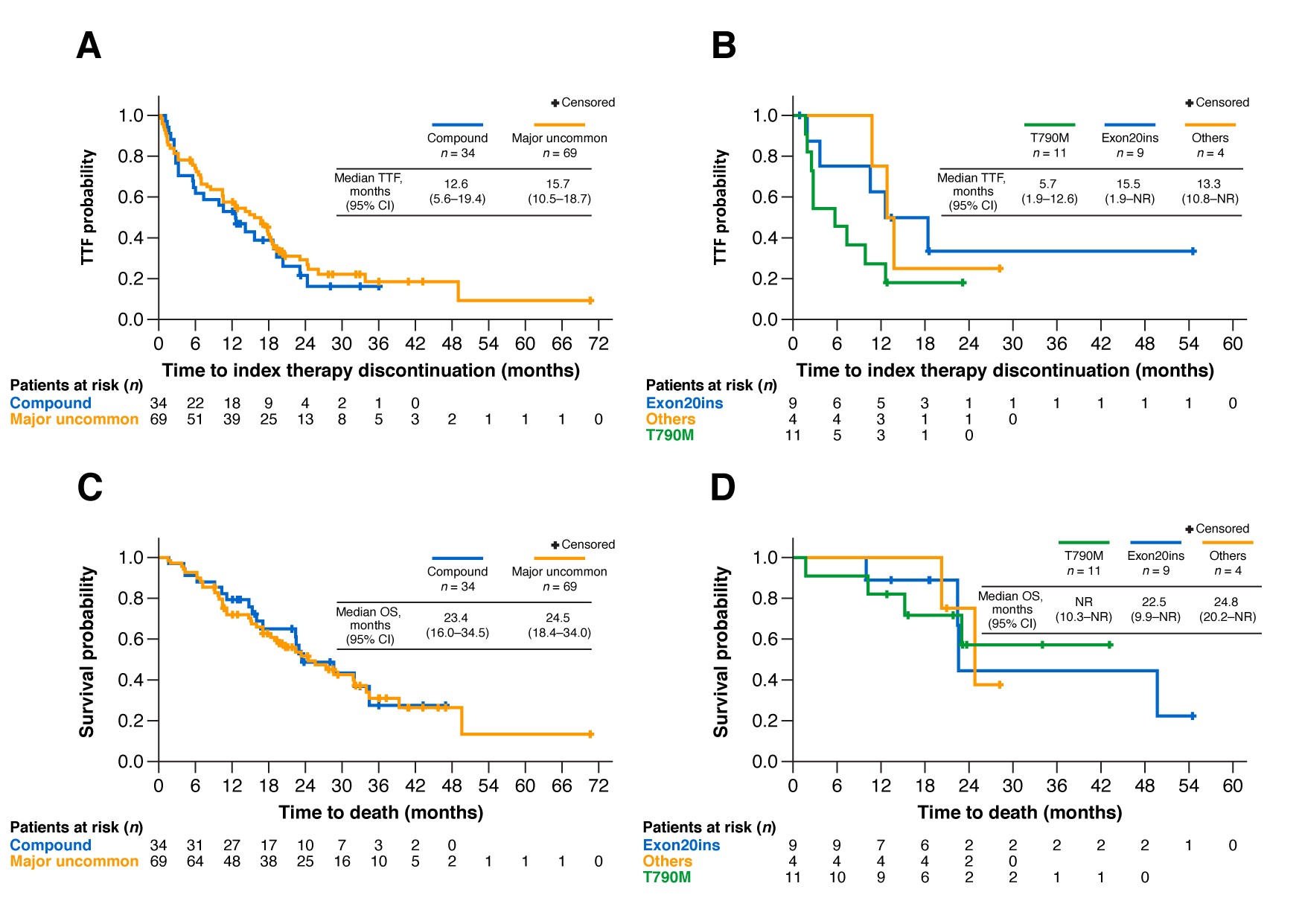

Supplement: oyac022_suppl_Supplementary_Material [file oyac022_suppl_supplementary_material.docx]
